# [^11^C]PIB amyloid quantification: effect of reference region selection

**DOI:** 10.1186/s13550-020-00714-1

**Published:** 2020-10-19

**Authors:** Fiona Heeman, Janine Hendriks, Isadora Lopes Alves, Rik Ossenkoppele, Nelleke Tolboom, Bart N. M. van Berckel, Adriaan A. Lammertsma, Maqsood Yaqub, on behalf of the AMYPAD Consortium

**Affiliations:** 1 Department of Radiology and Nuclear Medicine, Amsterdam Neuroscience, Amsterdam UMC, Vrije Universiteit Amsterdam, De Boelelaan 1117, 1081 HV Amsterdam, The Netherlands; 2 Neurology and Alzheimer Center, Amsterdam Neuroscience, Amsterdam UMC, Vrije Universiteit Amsterdam, De Boelelaan 1117, Amsterdam, The Netherlands; 3 Clinical Memory Research Unit, Lund University, Malmö, Sweden; 4 Imaging Division, Department of Radiology, University Medical Center Utrecht, Utrecht, The Netherlands

**Keywords:** [^11^C]PiB, Alzheimer’s disease, Amyloid PET, Quantification, Reference regions

## Abstract

**Background:**

The standard reference region (RR) for amyloid-beta (Aβ) PET studies is the cerebellar grey matter (GMCB), while alternative RRs have mostly been utilized without prior validation against the gold standard. This study compared five commonly used RRs to gold standard plasma input-based quantification using the GMCB.

**Methods:**

Thirteen subjects from a test–retest (TRT) study and 30 from a longitudinal study were retrospectively included (total: 17 Alzheimer’s disease, 13 mild cognitive impairment, 13 controls). Dynamic [^11^C]PiB PET (90 min) and T1-weighted MR scans were co-registered and time–activity curves were extracted for cortical target regions and the following RRs: GMCB, whole cerebellum (WCB), white matter brainstem/pons (WMBS), whole brainstem (WBS) and eroded subcortical white matter (WMES). A two-tissue reversible plasma input model (2T4k_V_b_) with GMCB as RR, reference Logan and the simplified reference tissue model were used to derive distribution volume ratios (DVRs), and standardized uptake value (SUV) ratios were calculated for 40–60 min and 60–90 min intervals. Parameter variability was evaluated using TRT scans, and correlations and agreements with the gold standard (DVR from 2T4k_V_b_ with GMCB RR) were also assessed. Next, longitudinal changes in SUVs (both intervals) were assessed for each RR. Finally, the ability to discriminate between visually Aβ positive and Aβ negative scans was assessed.

**Results:**

All RRs yielded stable TRT performance (max 5.1% variability), with WCB consistently showing lower variability. All approaches were able to discriminate between Aβ positive and Aβ negative scans, with highest effect sizes obtained for GMCB (range − 0.9 to − 0.7), followed by WCB (range − 0.8 to − 0.6). Furthermore, all approaches provided good correlations with the gold standard (*r* ≥ 0.78), while the highest bias (as assessed by the regression slope) was observed using WMES (range slope 0.52–0.67), followed by WBS (range slope 0.58–0.92) and WMBS (range slope 0.62–0.91). Finally, RR SUVs were stable across a period of 2.6 years for all except WBS and WMBS RRs (60–90 min interval).

**Conclusions:**

GMCB and WCB are considered the best RRs for quantifying amyloid burden using [^11^C]PiB PET.

## Background

Amyloid-beta accumulation (Aβ) in the brain is a pathological hallmark of Alzheimer’s disease (AD) and can be measured in vivo using positron emission tomography (PET) [1, 2]. One of the first amyloid PET tracers is Pittsburgh compound B ([^11^C]PiB), which binds with high specificity to fibrillar Aβ deposits [3, 4]. Both static and dynamic PET image acquisition protocols have been used, where the first is often preferred for routine and multi-centre studies due to its short duration and relatively simple processing. However, a static scan only provides a semi-quantitative measure of amyloid load, which can be affected by confounders [5–8]. Therefore, performing dynamic acquisitions and full quantification using kinetic modelling may be required for assessing subtle changes in amyloid load, which is of particular importance in longitudinal studies where other physiological parameters may change, thereby introducing bias [5]. In general, a disadvantage of such a protocol is the need for arterial sampling, which is logistically challenging, requires specially trained staff and dedicated equipment, and is particularly burdensome to the patient. A possible alternative to the use of arterial sampling is a reference tissue approach [9]. Reference tissue approaches rely on the assumption that a region devoid of specific binding, but otherwise having similar tissue characteristics as the target region of interest, is available (= reference region), providing an indirect input function and circumventing the need for arterial sampling [10, 11].

In case of imaging Aβ deposits in AD using [^11^C]PiB, the cerebellar grey matter (GMCB) meets the assumptions of a reference region in nearly all patients, and it has been validated against the plasma input approach [6, 12]. Only in rare familial forms and advanced stages of AD, this region might become compromised with Aβ plaques [13, 14]. In addition, accurate segmentation of this region can be challenging and may be hampered by truncation of the field of view in the lower portion of the brain. In recent years, several reports have proposed alternative reference regions, either aiming to overcome these issues, or aiming to improve effect sizes when measuring Aβ changes over time [15–17]. However, these alternative reference regions do not necessarily meet all requirements for a suitable reference tissue, such as having the same tissue characteristics as the target tissue or showing longitudinal stability and similar behaviour across diagnostic groups [15, 16]. One such region often used for amyloid quantification is whole cerebellum [16, 17]. Alternatively, reference tissues predominantly consisting of white matter, such as brainstem/pons or eroded subcortical white matter (centrum semiovale) have been proposed, in particular, for longitudinal amyloid quantification [17, 18]. However, age-related changes have been reported in the non-specific tracer retention of white matter regions, possibly compromising their use for longitudinal amyloid quantification [19].

To date, the impact of using alternative reference regions (RRs) on amyloid quantification has mainly been evaluated for semi-quantitative parameters [15–17]. Most alternative RRs have not been validated against the gold standard, i.e. full quantification with metabolite corrected plasma input curves or full quantification using a validated reference region.

Therefore, the present work focussed on the widely used [^11^C]PiB amyloid PET tracer and evaluated the use of the validated cerebellar grey matter as well as four alternative reference regions: whole cerebellum, white matter brainstem/pons, whole brainstem and eroded subcortical white matter. The performance of these regions was evaluated for both semi- and fully quantitative analysis in a test–retest (TRT) and longitudinal setting in terms of precision with respect to TRT variability, accuracy compared with the gold standard, stability over time (in case of the standardized uptake value, SUV), power for group discrimination and detecting physiologically plausible, longitudinal accumulation processes.

## Materials and methods

### Subjects

Clinical data of 43 participants belonging to two different studies, both conducted within the Amsterdam UMC, location VUmc, were included retrospectively [20, 21]. Thirteen subjects [6 cognitively unimpaired (CU), 1 mild cognitive impaired (MCI), 6 AD] were part of a TRT study and underwent arterial sampling, as described in detail by Tolboom et al. [21]. The other 30 subjects (11 CU, 12 MCI, 7 AD) were part of a longitudinal study as described by Ossenkoppele et al. [20]. In brief, all subjects received standard dementia screening for diagnostic purposes and amyloid PET scans were assessed visually (positive or negative) [21, 22]. Before enrolment, all participants provided written informed consent and the Medical Ethics Review Committee of the Amsterdam UMC, location VUmc, had approved both studies.

## Image acquisition

All subjects from the TRT study underwent a structural T1-weighted MR scan on a 1.5 T Siemens Sonata scanner (MPRAGE: matrix size 256 × 256 and 160 slices, voxel size 1.0 × 1.0 × 1.5 mm, echo time = 3.97 ms, repetition time = 2.700 ms, inversion time = 950 ms, flip angle 8°) and a test and same-day retest dynamic [^11^C]PiB PET scan (except for one subject) on a Siemens ECAT EXACT HR + scanner [21]. All participants first received a 10 min transmission scan for photon attenuation correction, followed by an intravenous [^11^C]PiB injection and simultaneously starting a 90 min dynamic PET scan [21]. Arterial blood was monitored continuously for the first 60 min using an online detection system and additional manual samples were drawn for calibration, to determine plasma to whole-blood ratios, and to measure plasma parent and metabolite fractions [21]. For seven subjects, arterial blood data were not available or not of sufficient quality for at least one of the scans. In addition, for one subject, the second scan was not used due to severe motion between PET frames. Consequently, a total of *N* = 6 test scans and *N* = 5 retest scans with plasma input data were available.

With respect to the longitudinal study, subjects also underwent similar T1-weighted MR and dynamic [^11^C]PiB PET scans at baseline, and follow-up (same scanners), 30.3 ± 5.4 (range 23–48) months later, but no arterial blood was sampled [20].

### Image processing

First, structural T1-weighted MR images were co-registered to their corresponding PET image. Next, PVE-lab software was used to segment grey matter (GM), white matter (WM) and cerebrospinal fluid (CSF), as well as to delineate volumes of interest (VOIs) based on the Hammers atlas [23, 24]. The following grey matter regions were used as target regions: medial and lateral anterior temporal lobe, posterior temporal lobe, superior, middle and inferior temporal gyrus, fusiform gyrus, parahippocampal and ambient gyrus, anterior and posterior cingulate gyrus, middle and orbitofrontal gyrus, gyrus rectus, inferior and superior frontal gyrus, pre- and post-central gyrus, superior parietal gyrus and the (infero)lateral remainder of the parietal lobe. In addition, a composite global cortical region was generated as the volume-weighted average across all target regions. The RRs included GMCB, whole cerebellum (WCB), white matter brainstem/pons (WMBS), whole brainstem (WBS) and the eroded subcortical white matter (WMES). The WMES was obtained by eroding the subject’s whole brain WM segmentation (using the *imerode* function in MATLAB) and manually removing cerebellar and brainstem white matter. Corresponding time–activity curves (TACs) were obtained by superimposing VOIs on the dynamic PET scan.

### Kinetic analysis

Only for scans where arterial plasma input data were available, the reversible two-tissue compartment model with four rate constants and additional blood volume fraction parameter (2T4k_V_b_) was used to estimate the volume of distribution (*V*_T_). Volume of distribution ratios (DVR_2T4k_Vb_ = *V*_T_ target / *V*_T_ reference) were calculated indirectly by using the validated GMCB as RR (here called: DVR_PI_GMCB_) (= gold standard) [6, 12].

For all scans, reference Logan (RLogan) was used to estimate DVR (DVR_RLOGAN_). The implementation did not require fixing *k*_2_′ (as per Eq. 7 from Logan et al. [25]) and a linearization time (t*) of 50 min p.i. was used [6, 25]. In addition, the simplified reference tissue model (SRTM) was used to estimate binding potential (*BP*_ND_) with parameter fit boundaries optimized per RR (see Additional file 1: Supplementary Table 1), and *BP*_ND_ + 1 (= DVR) was calculated for comparison [10]. Finally, standardized uptake value ratios (SUVr) were calculated for two frequently used acquisition windows (40–60 and 60–90 min p.i., SUVr_40−60_ and SUVr_60–90_, respectively) [6, 12]. For each reference tissue method, all RRs mentioned above were used.

**Table 1:** Subject demographics

TRT	CU (*N* = 6)	MCI (*N* = 1)	AD (*N* = 6)
Age	64.3 ± 5.7	71.0	61.0 ± 3.0
Females (%)	50%	100%	17%
MMSE	29.7 ± 0.5	28.0	20.7 ± 2.0

Longitudinal	CU (*N* = 11)	MCI (*N* = 12)	AD (*N* = 7)
Age	66.4 ± 7.3	67.4 ± 6.7	60.4 ± 5.4
Females (%)	27%	33%	14%
MMSE	29.4 ± 0.5	27.2 ± 2.5	25.3 ± 2.3

Values are depicted as *M* ± SD

### Statistical analysis

Statistical analyses were performed in IBM SPSS Statistics for Windows Version 24.0 (IBM Corp. Armonk New York U.S.A.), GraphPad Prism for Windows Version 7.04 (La Jolla California, USA), and Origin Version 2019b (OriginLab Corporation, Northampton, Massachusetts, USA). For each reference region and method, regional outliers were defined based on the median absolute deviation (MAD3) criterion assuming a non-normal distribution [26]. This resulted in a total of 36 values, across all subjects (from the 2T4k_V_b_ and SRTM models) being excluded from further analyses (see Additional file 1: supplementary materials for details). Differences in age and score on the Mini-Mental State Examination (MMSE) between diagnostic groups were assessed using nonparametric Kruskal–Wallis and post hoc Mann–Whitney *U* tests, while differences in the proportion of males and females were tested with Chi-square tests. As the TRT cohort consisted of only one MCI subject, this subject was not used for comparison.

#### Test–retest cohort

First, using the composite global cortical value, relative test–retest variability was calculated per RR and method according to Eq. 1, where the estimate of global cortical amyloid load (DVR or SUVr) of the test scan is denoted as T and for the retest scan as R

1 $${\text{TrT}}\;{\text{variability}} \, (\%) = \frac{{\left| {T - R} \right|}}{{0.5 \cdot \user2{ }\left| {T + R} \right|}} \cdot 100$$

Second, based on results obtained from the test scans (*N* = 6), agreement between regional quantification (for all RRs and reference tissue methods) and the gold standard (DVR_PI_GMCB_) was assessed using Bland–Altman (BA) analysis [27]. Next, linear regression analysis of the data points in the BA plots was used to assess whether (and to what extent) bias was dependent on underlying amyloid burden. Finally, correlations, slopes and intercepts between DVR_PI_GMCB_ and the corresponding parameter of interest derived from each of the RRs and methods were calculated using linear regression analysis.

#### Longitudinal cohort

A subset of subjects (*N* = 18) had information available on injected dose and patient weight, for which SUV TACs were calculated for all RRs. In addition, mean SUVs were calculated for all RRs and both acquisition windows (40–60 min and 60–90 min p.i.). The shape of the SUV TACs were assessed in the baseline scans, and the stability of the RRs over time was assessed using paired *t* tests with Bonferroni correction. Follow-up time was standardized to the average follow-up time across subjects (2.6 years) to account for between-subject differences.

Finally, as an exploratory analysis, the annual percentage change in the composite global cortical value was calculated per individual and for each of the RRs (according to Eq. 2)

2 $${\text{Annual}}\;{\text{percentage}}\;{\text{change}} = \frac{{{\text{FU}} - {\text{BL}}}}{{{\text{Years}}}} \cdot \frac{100}{{{\text{BL}}}}$$

With the parameter at follow-up scan as FU, at baseline scan as BL and Years stands for the number of years since baseline scan. These values were plotted against the baseline parameter and the relationship was assessed by fitting linear and quadratic models through the data. These models were chosen based on the previous literature and the known dose–response relationship of binding [1, 28, 29], where the hypothesis is that amyloid burden measured with PET plateaus at later stages of the disease [1]. Goodness of fit was assessed using the Akaike Information Criterion (AIC) [30].

#### Discriminative ability reference regions

For the global cortical parameter of interest, derived using each of the methods and RRs, the ability to discriminate between visually Aβ positive and Aβ negative scans was assessed using Mann–Whitney *U* tests with Bonferroni correction (using scans with stable longitudinal visual assessment: *N* = 80). In addition, the Hodges–Lehmann estimate of the median difference was used as measure of the effect size [31].

## Results

### Subjects

Demographics are presented in Table 1. As expected, CU subjects had higher MMSE scores (i.e. better global cognition) than AD subjects in both TRT (*p* = 0.003) and longitudinal (*p* = 0.001) studies. In addition, in the longitudinal study, higher MMSE scores were observed for CU compared with MCI subjects (*p* = 0.005), as well as a trend towards higher MMSE scores for MCI compared with AD subjects (*p* = 0.083). There were no differences with respect to age and sex.

### Test–retest cohort

#### Test–retest variability

**Fig. 1:**
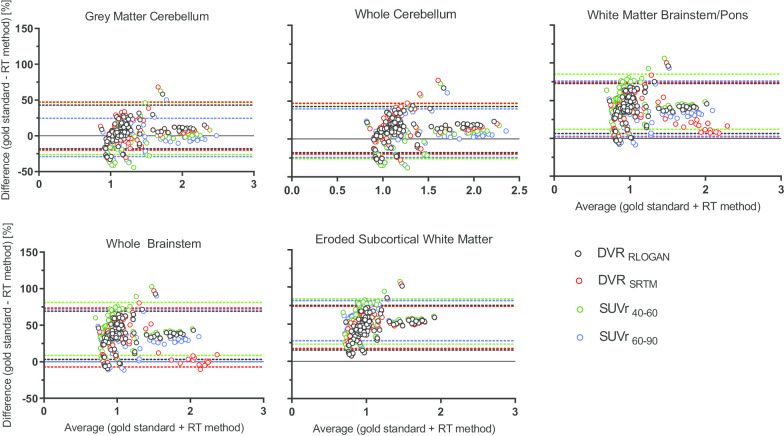
Bland–Altman: agreement with the gold standard for each reference region. Bland–Altman plot for each of the reference regions, showing the performance of all methods. RT: refers to the reference tissue method that is being compared

The maximum TRT variability across regions and methods was 5.1%, with lowest TRT variability observed for WCB across methods (Table 2). Across RRs, RLogan showed least variability overall, while SUVr_40−60_ showed less variability than SUVr_60–90_ (Table 2). Furthermore, the Bland–Altman analyses showed that for all RRs and methods, variability was most pronounced at low SUVR and DVR values (Fig. 1) and highest for the WMES (Additional file 1: Supplementary Table 2a).

**Table 2:** Relative test–retest variability across reference regions and methods

	DVR_RLOGAN_	DVR_SRTM_	SUVr_40−60_	SUVr_60−90_
GM cerebellum	2.8	2.9	3.5	5.1
Whole cerebellum	1.4	2.0	2.2	2.8
WM brainstem /pons	2.4	3.3	2.3	3.7
Whole brainstem	2.1	3.8	2.2	3.1
Subcortical eroded WM	2.4	2.7	3.7	3.9

All values are % TRT variability of global cortical averages for *N* = 12

Values depicted as mean (%) ± SD, MMSE = Mini-Mental State Examination

### Agreement with gold standard

Across methods, all RRs showed a strong correlation (*r* ≥ 0.78) with the gold standard, DVR_PI_GMCB_ (Table 3). Furthermore, GMCB and WCB RRs showed the smallest bias across methods as indicated by the regression slopes (Table 3, range 0.85–1.12, 0.81–1.05, respectively) and WMES the worst (Table 3, range 0.57–0.67) and shown by the Bland–Altman analysis (Fig. 1 and Additional file 1: Supplementary Table 2a). However, using RRs that contained white matter resulted in an underestimation compared with DVR_PI_GMCB_ for all parameters except SUVr’s calculated using the WCB (Table 3 and Fig. 1). In addition, the bias introduced by using WMES RR showed the strongest dependency on the underlying amyloid burden (Fig. 1 and Additional file 1: Supplementary Table 2b). Finally, across methods, SUVr_60−90_ showed a better correlation with DVR_PI_GMCB_ than SUVr_40−60_ (Table 3).

**Table 3:** Test–retest cohort: correlations between reference tissue methods with varying RRs and the gold standard: DVR_PI_GMCB_

Reference region	DVR_RLOGAN_	DVR_SRTM_	SUVr_40−60_	SUVr_60−90_
GM Cerebellum				
* r*	0.88	0.85	0.81	0.89
Slope	0.85	0.85	1.04	1.12
Intercept	0.14	0.18	− 0.03	− 0.10
Whole Cerebellum				
* r*	0.85	0.81	0.77	0.84
Slope	0.81	0.84	1.00	1.05
Intercept	0.09	0.03	− 0.11	− 0.15
WM Brainstem /Pons				
* r*	0.81	0.84	0.78	0.80
Slope	0.73	0.62	0.81	0.91
Intercept	− 0.07	0.04	− 0.24	− 0.30
Whole Brainstem Subcortical				
* r*	0.81	0.83	0.79	0.80
Slope	0.74	0.58	0.83	0.92
Intercept	− 0.06	0.11	− 0.24	− 0.29
Eroded WM				
* r*	0.83	0.86	0.82	0.86
Slope	0.57	0.52	0.67	0.63
Intercept	0.07	0.13	− 0.10	− 0.08

Values are shown for each of the methods and correspond to the linear regression analysis

### Longitudinal cohort

#### SUV reference region TACs and stability over time

**Fig. 2:**
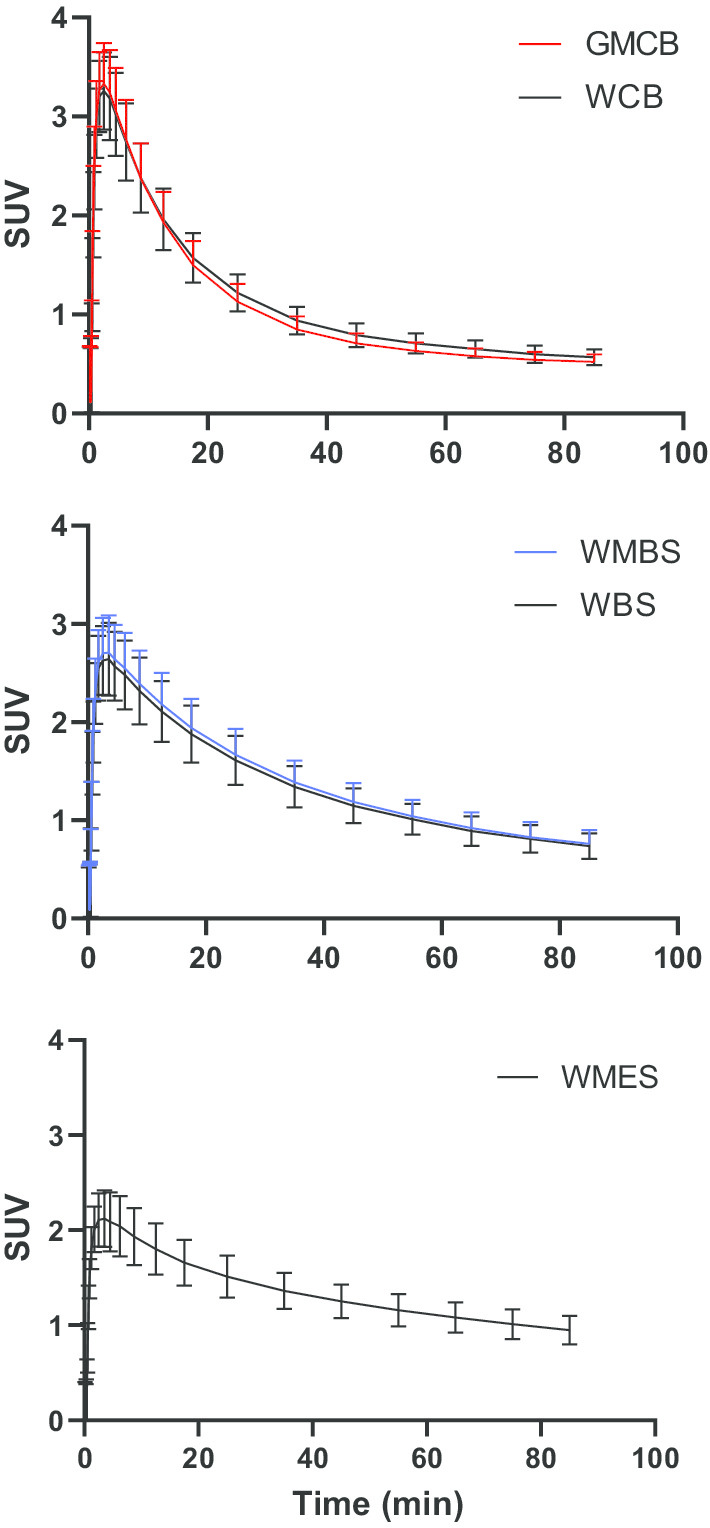
SUV TACs for all reference regions. Standardized uptake value time activity curves (corrected for weight and injected dose)

SUV TACs of the five RRs are depicted in Fig. 2, illustrating that WCB and GMCB, as well as WBS and WMBS showed a very similar shape. Furthermore, cerebellar RRs showed the steepest decline in uptake over time, followed by brainstem RRs and cerebellar and WMES RR TACs differed most.

With respect to the stability of longitudinal SUV uptake (60–90 min p.i.), significant decreases (after Bonferroni correction) between baseline and follow-up SUV measurements were only present for WBS and WMBS (*p* = 0.004, *p* = 0.003) and a trend level decrease was observed for WCB (*p* = 0.006). With respect to the early acquisition window (40–60 min p.i.), no significant differences were present, although the strongest trend was observed for WBS and WMBS.

#### Annual change and baseline amyloid load

**Fig. 3:**
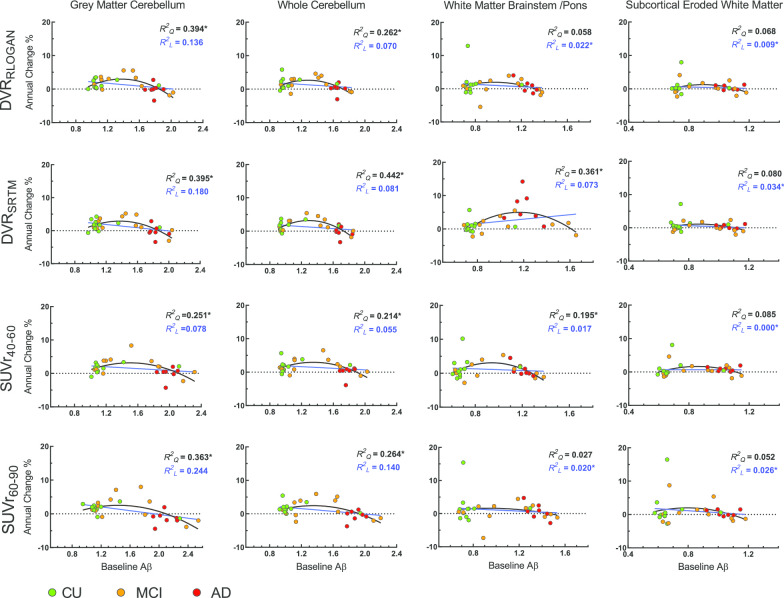
Baseline Aβ versus annual percentage change across reference regions and methods. The asterisk indicates the model that was preferred by the AIC

Across methods, the relationship between annual percentage change and baseline amyloid load as obtained by GMCB and WCB was best described by a quadratic relationship (Fig. 3) (ΔAIC GMCB RLogan: 8.0, SRTM: 6.4, SUVr_40−60_: 3.6, SUVr_60−90_: 2.4 and ΔAIC WCB RLogan: 4.2, SRTM: 12.3, SUVr_40−60_: 2.9, SUVr_60−90_: 2.0). In contrast, for WMBS and WBS the relationship was best described by a quadratic model for SRTM (ΔAIC: 8.5 and 12.2, respectively) and SUVr_40−60_ (ΔAIC: 3.3 and 3.8, respectively) and by a linear model for RLogan (ΔAIC: 1.6 and 1.5, respectively) and SUVr_60−90_ (ΔAIC: 2.4 and 2.5, respectively) (Fig. 3). Finally, with respect to WMES, the relationship was best described by a linear model for all methods (ΔAIC RLogan: 0.8, SRTM: 1.2 SUVr_40−60_: 0.0, SUVr_60−90_: 1.8).

### Scans from both cohorts

#### Discriminative ability reference regions

All parameters of interest derived using each of the RRs (and methods) were able to discriminate between Aβ positive and Aβ negative (*p* < 0.001) scans (Additional file 1: Supplementary Fig. 1). The highest effect sizes were obtained for GMCB (range − 0.9 to − 0.7), followed by the WCB RR (range − 0.8 to − 0.6) and lowest effect sizes for WMES (-0.4) (Additional file 1: Supplementary Table 3).

## Discussion

In the present [^11^C]PiB study, the performance of five reference regions was evaluated. All reference regions yielded relatively small test–retest variability and showed good correlations with the gold standard DVR_PI_GMCB_. However, largest bias, as shown by the regression slopes and BA analyses, was observed for white matter-based RRs. In addition, the choice of reference region did not impact the ability to differentiate between Aβ positive and negative scans, but the largest effect sizes were obtained for GMCB and WCB. Furthermore, the longitudinal study showed that SUV changed over time for both WBS and WMBS RRs, but only when using the late acquisition window (60−90 min). Finally, the relationship between baseline amyloid and Aβ accumulation was best described by a quadratic model, as expected, for GMCB and WCB.

While the maximum TRT variability was 5.1% across methods, the WCB RR showed consistently lower variability (Table 2). This may be related to the fact that this region is less prone to segmentation errors than for example GMCB and has more counts compared with the brainstem as a result of its larger volume. In addition, WCB may also outperform WMES in terms of TRT variability because the latter showed bias that was more dependent on the underlying amyloid burden (Fig. 1 and Additional file 1: Supplementary Table 2b). Finally, all regional parameters of interest, derived using all methods and RRs, showed good correlations (*r ≥ *0.78) with regional DVR_PI_GMCB_ (Table 3).

Using GMCB and WCB as RR yielded, as expected, least bias as compared with the gold standard (as shown by the linear regression: Table 3 and BA analysis: Fig. 1 and Additional file 1: Supplementary Table 2a). RRs that primarily contained white matter showed substantial underestimation compared with values obtained by the plasma input model, except for WCB, were this underestimation was only observed for RLogan and SRTM (Table 3). This underestimation is likely a result of both the relatively high uptake in white compared with grey matter and the different kinetics in this tissue compared to other RRs as illustrated by Fig. 2. Furthermore, given that the two cerebellar as well as the two brainstem RR SUV TACs were very similar in shape, relatively small differences in performance with respect to precision and accuracy were expected. These findings also indicate that the effect of choice of tissue of the RR on quantification is smaller than the effect of using a different anatomical RR. Furthermore, for WMES RR, bias (as shown by the BA analysis) was most dependent on the underlying amyloid burden (Additional file 1: Supplementary Table 2b). Therefore, using WMES for normalization purposes could be problematic, in particular, for analysing regions or subjects spanning the AD continuum.

The longitudinal results showed significant decreases in WBS and WMBS SUV only for the late (60–90 min) acquisition window. However, a similar trend (although not significant) was present for the early (40–60 min) acquisition window. This finding might be related to the fact that SUV does not take flow changes into account [20]. As such, using WBS or WMBS for normalization purposes, may result in an overestimation of the true Aβ load and this would be particularly problematic for longitudinal Aβ quantification [19]. In fact, effects of these confounding factors may explain why some studies have reported increased power for detecting longitudinal changes or larger between group differences in rates of Aβ change, using white matter RRs [32, 33]. Moreover, decreases in white matter SUV also may explain the lower pons and WMES SUVR values for groups of increasing disease severity (using GMCB as RR), as previously reported by Tryputsen and colleagues [34], although the authors themselves provide a different explanation by suggesting it could be due to increasing GMCB Aβ load. Ideally, one would have used *V*_T_ for assessing the stability of RRs over time, but this was not possible as these subjects did not undergo arterial sampling.

Furthermore, results showed that although all RRs were able to discriminate between Aβ positive and Aβ negative scans, GMCB and WCB provided the highest effect sizes, while WMES provided the poorest results. Therefore, GMCB and WCB would be preferred for detecting more subtle between-group differences. These findings partially differ from some previous reports, likely due to differences in study population, study design or criteria used for defining the optimal RR. For example, some studies reported highest effect sizes for GMCB and pons or for WMES and pons when discriminating between diagnostic groups, which only partly agrees with the present results when discriminating between Aβ positive and Aβ negative scans [35, 36]. Moreover, the high effect sizes reported for WM RRs could also be related to the effect of confounding factors, as discussed above. It should be noted, however, that these results belong to a group classification analysis, and hence they cannot be compared directly with findings from studies assessing the statistical power for detecting longitudinal changes in Aβ burden, that employ a within-subject design [32, 34]. Finally, differences in the criteria used for identifying the optimal RR can have a significant impact on outcome. For example, while Schwarz and colleagues exclusively focused on longitudinal criteria to recommend a combination of voxels from supratentorial white matter and whole cerebellum as RR, the present study used a combination of criteria based on a comparison against the gold standard, test–retest variability and longitudinal performance [33].

In the present study, an inverted u-shaped relationship between baseline amyloid load and Aβ accumulation was observed only for GMCB and WCB RRs. This pattern has been reported previously [28, 37, 38], and is in line with the known sigmoidal dose response relationship of binding [29]. It should be noted that this was only an exploratory analysis, and further studies are needed to explore this relationship and possible between group differences in Aβ accumulation (e.g. by diagnostic or Aβ status) in a larger dataset.

Taken together, the present results suggest that GMCB and WCB are suitable RRs with respect to analysing [^11^C]PiB scans. Overall, accuracy as compared with the gold standard was higher using GMCB, while precision (as assessed by measurement variability and dependency of the bias on underlying Aβ burden) was more favourable using WCB. Therefore, in cross-sectional studies one might prefer GMCB, as it more closely adheres to the “truth”, while in longitudinal studies, where stability of results outweighs a small bias, WCB would be preferred. Finally, it is important to note that the results of the present study relate to [^11^C]PiB and are not necessarily translatable to other tracers. As shown previously by Villemagne and colleagues for SUVr, the most stable RR may differ per tracer [39], and this finding was supported by studies using both [^18^F]florbetaben and [^18^F]florbetapir [16, 40]. These between-tracer discrepancies may be the result of differences in non-specific binding in the reference region (as compared with the F-18 labelled tracers) or violations of the reference tissue approach. Hence, they emphasize the importance of a per tracer evaluation of suitable RRs.

## Conclusion

Outcome measures of all reference regions correlated well with the gold standard and showed stable test–retest performance. However, the largest bias compared with the gold standard was observed for eroded subcortical white matter, followed by whole brain stem and white matter brainstem/pons. Furthermore, using the 60–90-min acquisition window, significant longitudinal alterations in SUV were observed, for whole brain stem and white matter brainstem/pons reference regions. Therefore, grey matter cerebellum and whole cerebellum are considered to be the best RRs for measuring amyloid burden with [^11^C]PiB.

## Supplementary information

**Additional file 1**. Supplementary materials [^11^C]PiB amyloid quantification: effect of reference region selection.

## Data Availability

The data used in this study can be made available upon reasonable request.

## Abbreviations

RR: Reference region
Aβ: Amyloid-beta
PET: Positron emission tomography
GMCB: Cerebellar grey matter
TRT: Test–retest
AD: Alzheimer’s disease
MCI: Mild cognitive impairment
PiB: Pittsburgh compound B
^11^C: Carbon-11
MR: Magnetic resonance
TACs: Time–activity curves
WCB: Whole cerebellum
WMBS: White matter brainstem/pons
WBS: Whole brainstem
WMES: Eroded subcortical white matter
SRTM: Simplified reference tissue method
RLogan: Reference Logan
DVR: Distribution volume ratio
PI: Plasma input
*BP*_ND_: Non-displaceable binding potential
SUV: Standardized uptake value
SUVR: Standardized uptake value ratio
VOI: Volume of interest
2T4k_Vb: Reversible two-tissue compartment model (4 rate constants) with additional blood volume fraction parameter
COV: Coefficient of variation
GM: Grey matter
WM: White matter
CSF: Cerebrospinal fluid
*V*_T_: Volume of distribution
MAD3: Median absolute deviation
MMSE: Mini-Mental State Examination
FU: Follow-up
BL: Baseline scan
AIC: Akaike information criterion
BA: Bland–Altman

## References

[1] Jack CR, Knopman DS, Jagust WJ, Shaw LM, Aisen PS, Weiner MW (2010). Hypothetical model of dynamic biomarkers of the Alzheimer’s pathological cascade. Lancet Neurol.

[2] Mallik A, Drzezga A, Minoshima S (2017). Clinical amyloid imaging. Semin Nucl Med.

[3] Mathis CA, Wang Y, Holt DP, Huang G-F, Debnath ML, Klunk WE (2003). Synthesis and evaluation of 11C-labeled 6-substituted 2-arylbenzothiazoles as amyloid imaging agents. J Med Chem.

[4] Rabinovici GD, Jagust WJ (2009). Amyloid imaging in aging and dementia: testing the amyloid hypothesis in vivo. Behav Neurol.

[5] van Berckel BNM, Ossenkoppele R, Tolboom N, Yaqub M, Foster-Dingley JC, Windhorst AD (2013). Longitudinal amyloid imaging using 11C-PiB: methodologic considerations. J Nucl Med.

[6] Yaqub M, Tolboom N, Boellaard R, van Berckel BNM, van Tilburg EW, Luurtsema G (2008). Simplified parametric methods for [11C]PIB studies. Neuroimage.

[7] Carson RE, Channing MA, Blasberg RG, Dunn BB, Cohen RM, Rice KC (1993). Comparison of bolus and infusion methods for receptor quantitation: application to [18 F]cyclofoxy and positron emission tomography. J Cereb Blood Flow Metab.

[8] Heeman F, Yaqub M, Alves IL, Heurling K, Bullich S, Gispert JD, et al. Simulating the effect of cerebral blood flow changes on regional quantification of [18F]flutemetamol and [18F]florbetaben studies: J Cereb Flow Metab. 2020. https://journals.sagepub.com/doi/10.1177/0271678X20918029 10.1177/0271678X20918029 PMC7907983 32281514

[9] Gunn RN, Gunn SR, Cunningham VJ (2001). Positron emission tomography compartmental models. J Cereb Blood Flow Metab.

[10] Lammertsma AA, Hume SP (1996). Simplified reference tissue model for PET receptor studies. Neuroimage.

[11] Cunningham VJ, Hume SP, Price GR, Ahier RG, Cremer JE, Jones AKP (1991). Compartmental analysis of diprenorphine binding to opiate receptors in the rat in vivo and its comparison with equilibrium data in vitro. J Cereb Blood Flow Metab.

[12] Price JC, Klunk WE, Lopresti BJ, Lu X, Hoge JA, Ziolko SK (2005). Kinetic modeling of amyloid binding in humans using PET imaging and pittsburgh compound-B. J Cereb Blood Flow Metab.

[13] Thal DR, Rüb U, Orantes M, Braak H (2002). Phases of Aβ-deposition in the human brain and its relevance for the development of AD. Neurology.

[14] Wegiel J, Wisniewski HM, Dziewiatkowski J, Badmajew E, Tarnawski M, Reisberg B (1999). Cerebellar atrophy in Alzheimer’s disease—clinicopathological correlations. Brain Res.

[15] Edison P, Hinz R, Ramlackhansingh A, Thomas J, Gelosa G, Archer HA (2012). Can target-to-pons ratio be used as a reliable method for the analysis of [11C]PIB brain scans?. NeuroImage.

[16] Bullich S, Villemagne VL, Catafau AM, Jovalekic A, Koglin N, Rowe CC (2017). Optimal reference region to measure longitudinal amyloid-β change with 18F-florbetaben PET. J Nucl Med.

[17] Landau SM, Fero A, Baker SL, Koeppe R, Mintun M, Chen K (2015). Measurement of longitudinal β-amyloid change with 18F-florbetapir PET and standardized uptake value ratios. J Nucl Med.

[18] Klunk WE, Koeppe RA, Price JC, Benzinger TL, Devous MD, Jagust WJ (2015). The centiloid project: standardizing quantitative amyloid plaque estimation by PET. Alzheimer’s & Dement.

[19] Lowe VJ, Lundt ES, Senjem ML, Schwarz CG, Min H-K, Przybelski SA (2018). White matter reference region in PET studies of 11C-pittsburgh compound B uptake: effects of age and amyloid-β deposition. J Nucl Med.

[20] Ossenkoppele R, Tolboom N, Foster-Dingley JC, Adriaanse SF, Boellaard R, Yaqub M (2012). Longitudinal imaging of Alzheimer pathology using [11C]PIB, [18F]FDDNP and [18F]FDG PET. Eur J Nucl Med Mol Imaging.

[21] Tolboom N, Yaqub M, Boellaard R, Luurtsema G, Windhorst AD, Scheltens P (2009). Test-retest variability of quantitative [11C]PIB studies in Alzheimer’s disease. Eur J Nucl Med Mol Imaging.

[22] Ossenkoppele R, Prins ND, Pijnenburg YAL, Lemstra AW, van der Flier WM, Adriaanse SF (2013). Impact of molecular imaging on the diagnostic process in a memory clinic. Alzheimers Dement.

[23] Rask T, Dyrby T, Comerci M, Alfano B, Quarantelli M, Berkouk K, et al. PVElab: Software for correction of functional images for partial volume errors. Neuroimage. 2004. https://kar.kent.ac.uk/27739/

[24] Hammers A, Allom R, Koepp MJ, Free SL, Myers R, Lemieux L (2003). Three-dimensional maximum probability atlas of the human brain, with particular reference to the temporal lobe. Hum Brain Mapp.

[25] Logan J, Fowler JS, Volkow ND, Wang GJ, Ding YS, Alexoff DL (1996). Distribution volume ratios without blood sampling from graphical analysis of PET data. J Cereb Blood Flow Metab.

[26] Leys C, Ley C, Klein O, Bernard P, Licata L (2013). Detecting outliers: do not use standard deviation around the mean, use absolute deviation around the median. J Exp Soc Psychol.

[27] Martin BJ, Altman DG (1986). Statistical methods for assessing agreement between two methods of clinical measurement. The Lancet..

[28] Jack CR, Wiste HJ, Lesnick TG, Weigand SD, Knopman DS, Vemuri P (2013). Brain b-amyloid load approaches a plateau. Neurology.

[29] Page SW, Maddison JE. Chapter 1—principles of clinical pharmacology. In: Maddison JE, Page SW, Church DB, Maddison JE, Page SW, Church DB (eds). Small animal clinical pharmacology, 2nd edn). Edinburgh; 2008 [cited 2019 Nov 11]. p. 1–26. https://www.sciencedirect.com/science/article/pii/B9780702028588500038

[30] Akaike H (1974). A new look at the statistical model identification. IEEE Trans Autom Control.

[31] Hodges JL, Lehmann EL (1963). Estimates of location based on rank tests. Ann Math Statist.

[32] Su Y, Blazey TM, Owen CJ, Christensen JJ, Friedrichsen K, Joseph-Mathurin N, et al. Quantitative amyloid imaging in autosomal dominant Alzheimer’s disease: results from the DIAN Study Group. Herholz K, editor. PLoS ONE. 2016;11:e0152082. 10.1371/journal.pone.0152082 PMC4807073 27010959

[33] Schwarz CG, Senjem ML, Gunter JL, Tosakulwong N, Weigand SD, Kemp BJ (2017). Optimizing PiB-PET SUVR change-over-time measurement by a large-scale analysis of longitudinal reliability, plausibility, separability, and correlation with MMSE. Neuroimage.

[34] Tryputsen V, DiBernardo A, Samtani M, Novak GP, Narayan VA, Raghavan N (2015). Optimizing regions-of-interest composites for capturing treatment effects on brain amyloid in clinical trials. J Alzheimer’s Dis IOS Press.

[35] Oliveira F, Leuzy A, Castelhano J, Chiotis K, Hasselbalch SG, Rinne J (2018). Data driven diagnostic classification in Alzheimer’s disease based on different reference regions for normalization of PiB-PET images and correlation with CSF concentrations of Aβ species. NeuroImage Clin.

[36] Yun HJ, Moon SH, Kim HJ, Lockhart SN, Choe YS, Lee KH, et al. Centiloid method evaluation for amyloid PET of subcortical vascular dementia. Sci Rep; 2017;7:16322. 10.1038/s41598-017-16236-1 PMC5701176 29176753

[37] Villemagne VL, Burnham S, Bourgeat P, Brown B, Ellis KA, Salvado O (2013). Amyloid β deposition, neurodegeneration, and cognitive decline in sporadic Alzheimer’s disease: a prospective cohort study. Lancet Neurol.

[38] Leal SL, Lockhart SN, Maass A, Bell RK, Jagust WJ (2018). Subthreshold amyloid predicts tau deposition in aging. J Neurosci.

[39] Villemagne VL, Bourgeat P, Doré V, Macaulay L, Williams R, Ames D (2015). Amyloid imaging in therapeutic trials: the quest for the optimal reference region. Alzheimer’s Dement.

[40] Ottoy J, Verhaeghe J, Niemantsverdriet E, Wyffels L, Somers C, Roeck ED (2017). Validation of the semiquantitative static SUVR method for 18F-AV45 PET by pharmacokinetic modeling with an arterial input function. J Nucl Med.

