# Supplementary material for: [11C]PIB amyloid quantification: effect of reference region selection
Source: EJNMMI Res. 2020 Oct 19;10:123. doi: 10.1186/s13550-020-00714-1 (PMC7572969; doi:10.1186/s13550-020-00714-1)
Supplement: Supplementary file 1 — Additional file 1. Supplementary materials [11C]PiB amyloid quantification: effect of reference region selection. [file 13550_2020_714_MOESM1_ESM.docx]

**Supplementary materials**

**Validation parameter fit boundaries**

All data were fitted using SRTM with parameter fit boundaries based on theoretical assumptions, considering grey and white matter reference regions were used. Lower boundaries were the following: *R*_1_: 10^-7^, *k*_2_: 10^-7^, *BP*_ND_: -1, and upper boundaries: *R*_1_: 10^2^, *k*_2_: 10^2^, *BP*_ND_: 10^2^. To optimise the lower and upper boundaries, the minimum and maximum parameter values of a clean data-set were used. This data-set was created by removing all regional fit parameters with a standard deviation (SD) >50%, i.e. if *BP*_ND_ SD >50%, also the corresponding *R*_1_ and *k*_2_ values were removed (see Supplementary Table 1 below).

**Supplementary Table 1.**

**Parameter fit boundaries optimised per reference region**

|  | ***R*_1_** | ***k*_2_** | ***BP*_ND_** |
| --- | --- | --- | --- |
| **GM Cerebellum** | 0.500 | 0.011 | -0.200 |
|  | 1.100 | 0.400 | 1.600 |
| **Whole Cerebellum** | 0.550 | 0.014 | -0.200 |
|  | 1.200 | 0.900 | 1.350 |
| **WM Brainstem/ Pons** | 0.700 | 0.011 | -0.500 |
|  | 1.550 | 2.200 | 1.100 |
| **Whole Brainstem** | 0.700 | 0.009 | -0.450 |
|  | 1.800 | 3.800 | 1.250 |
| **Subcortical Eroded** | 0.800 | 0.011 | -0.500 |
| **WM** | 2.150 | 1.350 | 0.400 |

**Regional outliers**

Outliers were observed for the following regions: fusiform gyrus (2), anterior (13) and posterior cingulate gyrus (5), gyrus rectus (3), inferior frontal gyrus (2), inferior lateral remainder of parietal lobe (1), middle and inferior temporal gyrus (1), middle frontal gyrus (3), orbitofrontal gyrus (2), superior frontal gyrus (2), superior parietal gyrus (1), superior temporal gyrus (1).

**Supplementary Table 2a.**

**Bland-Altman: agreement with the gold standard for all reference tissue methods with varying RRs**

|  | | **DVR_RLOGAN_** | **DVR_SRTM_** | **SUVr_40-60_** | **SUVr_60-90_** |
| --- | --- | --- | --- | --- | --- |
| **GM Cerebellum** | Bias | 4.7 | 3.3 | -0.5 | -2.2 |
|  | 95% CI | [-22.7 32.0] | [-27.1 33.7] | [-34.0 33.0] | [-29.1 24.6] |
| **Whole Cerebellum** | Bias | 12.4 | 13.7 | 10.3 | 7.7 |
|  | 95% CI | [-18.1 43.0] | [-19.9 47.3] | [-26.0 46.6] | [-24.4 39.8] |
| **WM Brainstem/Pons** | Bias | 39.7 | 38.3 | 48.8 | 39.6 |
|  | 95% CI | [6.6 72.7] | [2.2 74.3] | [12.3 85.3] | [3.0 76.2] |
| **Whole Brainstem** | Bias | 36.3 | 33.3 | 45.0 | 36.2 |
|  | 95% CI | [3.3 69.2] | [-7.0 73.6] | [8.7 81.3] | [0.0 72.3] |
| **Subcortical Eroded WM** | Bias | 45.4 | 45.8 | 53.7 | 54.7 |
|  | 95% CI | [15.3 75.4] | [17.5 74.1] | [23.3 84.1] | [27.7 81.8] |

Bias: mean % bias as compared with the gold standard, DVR_PI_GMCB_ and 95% confidence interval (CI) calculated as follows: ((gold standard – RT method)/average*100). Data used: 18 target tissue regions derived from six subjects with plasma input data available.

**Supplementary Table 2b.**

**Relationship between bias (%) and underlying amyloid burden**

|  | **DVR_RLOGAN_** | **DVR_SRTM_** | | **SUVr_40-60_** | | **SUVr_60-90_** |  |
| --- | --- | --- | --- | --- | --- | --- | --- |
| **GM Cerebellum** | 15.6^#^ | | 12.5^#^ | | 9.2* | 3.4 | |
| **Whole Cerebellum** | 16.1^#^ | | 11.3* | | 7.8 | 3.0 | |
| **WM Brainstem/Pons** | 11.7* | | -22.6^#^ | | 0.1 | -3.3 | |
| **Whole Brainstem** | 11.0* | | -14.7^#^ | | -0.2 | -4.3 | |
| **Subcortical Eroded WM** | 25.4^#^ | | 22.8^#^ | | 13.2* | 9.9* | |

Values correspond to slopes of the linear regression analysis through Bland-Altman data-points.

Data used: 18 target tissue regions derived from six subjects with plasma input data available.

* *p*<0.05, ^#^ *p*<0.01

**Supplementary Figure 1. Discriminating between Aβ+ and Aβ- scans**

Individual global cortical averages were used. Plots show the mean and standard deviation per RR for each of the methods.

**Supplementary Table 3.**

**Hodges-Lehmann estimate of the median difference between Aβ+ and Aβ- scans**

|  | | **DVR_RLOGAN_** | **DVR_SRTM_** | **SUVr_40-60_** | **SUVr_60-90_** |
| --- | --- | --- | --- | --- | --- |
| **GM Cerebellum** | Estimate | -0.7 | -0.7 | -0.8 | -0.9 |
|  | LB UB | [-0.7 -0.6] | [-0.7 -0.6] | [-0.9 -0.8] | [-1.0 -0.7] |
| **Whole Cerebellum** | Estimate | -0.6 | -0.7 | -0.8 | -0.8 |
|  | LB UB | [-0.7 -0.6] | [-0.7 -0.6] | [-0.8 -0.7] | [-0.9 -0.7] |
| **WM Brainstem/Pons** | Estimate | -0.5 | -0.5 | -0.6 | -0.6 |
|  | LB UB | [-0.5 -0.5] | [-0.7 -0.4] | [-0.6 -0.5] | [-0.7 -0.6] |
| **Whole Brainstem** | Estimate | -0.5 | -0.6 | -0.6 | -0.6 |
|  | LB UB | [-0.6 -0.5] | [-0.8 -0.5] | [-0.6 -0.5] | [-0.7 -0.6] |
| **Subcortical Eroded WM** | Estimate | -0.4 | -0.4 | -0.4 | -0.4 |
|  | LB UB | [-0.4 -0.3] | [-0.4 -0.3] | [-0.4 -0.4] | [-0.4 -0.4] |

Hodges-Lehmann estimate and its lower and upper boundaries (LB, UB). As null is not within the boundaries, the null-hypothesis of no differences between Aβ+ and Aβ- scans is rejected. All global cortical values belong to baseline scans from the test-retest and longitudinal study (*N*=43)
